# Assessment scale of risk for surgical positioning injuries**^1^**

**DOI:** 10.1590/1518-8345.0644.2704

**Published:** 2016-08-29

**Authors:** Camila Mendonça de Moraes Lopes, Vanderlei José Haas, Rosana Aparecida Spadoti Dantas, Cheila Gonçalves de Oliveira, Cristina Maria Galvão

**Affiliations:** 2 PhD, Adjunct Professor, Universidade Federal do Rio de Janeiro, Macaé, RJ, Brazil.; 3 Professor, Universidade Federal do Triângulo Mineiro, Uberaba, MG, Brazil.; 4 Associate Professor, Escola de Enfermagem de Ribeirão Preto, Universidade de São Paulo, PAHO/WHO Collaborating Centre for Nursing Research Development, Ribeirão Preto, SP, Brazil.; 5 Specialist in Biomedical Engineering and Clinical Engineering.; 6 Full Professor, Escola de Enfermagem de Ribeirão Preto, Universidade de São Paulo, PAHO/WHO Collaborating Centre for Nursing Research Development, Ribeirão Preto, SP, Brazil.

**Keywords:** Perioperative Nursing, Intraoperative Period, Nursing Care, Wounds and Injuries, Risk Assessment, Patient Positioning

## Abstract

**Objective:**

to build and validate a scale to assess the risk of surgical positioning injuries in adult patients.

**Method:**

methodological research, conducted in two phases: construction and face and content validation of the scale and field research, involving 115 patients.

**Results:**

the Risk Assessment Scale for the Development of Injuries due to Surgical Positioning contains seven items, each of which presents five subitems. The scale score ranges between seven and 35 points in which, the higher the score, the higher the patient's risk. The Content Validity Index of the scale corresponded to 0.88. The application of Student's t-test for equality of means revealed the concurrent criterion validity between the scores on the Braden scale and the constructed scale. To assess the predictive criterion validity, the association was tested between the presence of pain deriving from surgical positioning and the development of pressure ulcer, using the score on the Risk Assessment Scale for the Development of Injuries due to Surgical Positioning (p<0.001). The interrater reliability was verified using the intraclass correlation coefficient, equal to 0.99 (p<0.001).

**Conclusion:**

the scale is a valid and reliable tool, but further research is needed to assess its use in clinical practice.

## Introduction

Surgical positioning is a relevant procedure, executed by all professional involved in patient care (nursing, anesthetic and surgical team) during the intraoperative period. Therefore, the patient's particularities should be taken into account, as well as the surgeon's preferences for the best exposure of the surgical site, the surgical technique to be applied and access needed for medication administration and the patient's monitoring and ventilation by the anesthesist. Hence, the implementation of interventions based on recent evidence is crucial to guarantee safe and comfortable surgical positioning, with a view to preventing complications in the tegumentary, neurological, vascular and respiratory systems^1^ ^-^ ^3^.

In this context, perioperative nurses' activities are fundamental. These professionals should be familiar with the anatomic and physiological alterations the surgical positioning and the equipment and devices available to be of help in the execution of the procedure cause in the patient's organism, permitting the planning and implementation of effective interventions to guarantee the prevention of complications that can arise due to the prolonged stay of the patient in each type of surgical position^4^.

The main complications related to surgical positioning include musculoskeletal pain, skin and peripheral nerve injuries and compartment syndrome^4^. Studies have been published in the literature about the occurrence of injuries deriving from surgical positioning. In a descriptive study to identify the risk factors for the emergence of this type of lesion, the results evidenced that, in the research sample (n=50), 74% of the patients were affected by pressure ulcer (stage I)^5^.

In another study, the results indicated that, out of 172 participants, 12.2% were affected by surgical positioning injuries, and five patients presented more than one type of injury (26 injuries in total), that is: 9.9% patients referred severe pain in pressure points, 4.7% suffered peripheral nerve injuries and 0.6% erythema^6^.

In the literature, there is a lack of data on the incidence of peripheral nerve injuries due to surgical positioning. In a descriptive study, including 2,304 patients submitted to colorectal surgery, 0.3% presented this type of injury, five of whom underwent open surgery and three minimally invasive procedures (videolaparoscopy)^7^.

At the health services, the use of a risk assessment scale can help the nurse to identify factors predisposing to the development of injuries and the implementation of prevention measures and, consequently, to the improvement of health care^8^.

To offer support through research that contributes to improve care delivery to surgical patients, in this study, the objective was to construct and validate a risk assessment scale for surgical positioning injuries in adult patients.

## Method

A methodological research was conducted in two phases: construction and face and content validation of the Risk Assessment Scale for the Development of Injuries due to Surgical Positioning (ELPO) and field research to analyze the validity and reliability measures of the proposed scale.

Based on the results of the integrative review the authors undertook in the Master's program, a periodical search for new studies on care related to the patient's surgical positioning, as well as accumulated professional experience, the domains of the ELPO were defined (version 1). That version contained seven items, each of which contained five subitems, organized according to the anatomous and physiological implications of the surgical positions on the patient's body. The proposed scale contained the following items: type of surgical position, duration of surgery, type of anesthesia, support surface, limb position, comorbidities and patient age.

Invited experts (n=30) undertook the face and content validation of the scale. They were selected through the Lattes Platform and complied with the following criteria: nurse, holding a Ph.D., knowledge area perioperative nursing, research development on risks and complications deriving from surgical anesthesia and related to the theme surgical positioning of the patient.

Using an online tool, the invited experts accessed the ELPO (version 1) and the assessment questionnaire for this validation phase. The questionnaire contained the scale items with a short theoretical explanation and the respective subitems with the following alternative replies: I completely disagree; I disagree: I neither disagree nor agree; I agree; I completely agree (Likert scale ranging from 1 to 5). At the end of the assessment of each item, space was provided for the expert to comment if necessary. The experts suggested: indicating the duration of the surgery in a closed interval to impede mistaken interpretation and, in the item limb position, the change of the term body alignment to anatomic position, which the researchers accepted. The experts undertook the face and content validation in the first semester of 2012.

The field research was undertaken to analyze the concurrent criterion validity (comparison between Braden scale score and ELPO score), the predictive criterion validity (assessment of pain outcomes deriving from surgical positioning and occurrence of pressure ulcer in postoperative patients using ELPO score) and inter-rater reliability assessment (application of ELPO during intraoperative period by two professionals at the same time).

The data were collected at a general medium-sized hospital in the South of the State of Minas Gerais. The target population consisted of male and female patients submitted to surgical procedures in any surgical specialty. As regards the inclusion criterion, patients should be 18 years of age or older and be submitted to elective surgery.

To calculate the sample size for the inter-rater reliability analysis, an expected Inter-rater Correlation Coefficient of ICC=0.7 was considered between the safety scores. The ICC should not be inferior to 0.5, with a test power of 90% and a significance level of α=0.05. Using the software PASS 2002 (Power Analysis and Sample Size) and these *a priori* coefficients, the minimum sample size was 87 subjects. A convenience sample was used and 115 patients participated in the field research in accordance with the inclusion criteria.

To collect the data, besides the application of the ELPO (version 2), the following tools were used: tool 1 (built by the researchers) to register pre and postoperative information, which contained data on the patient identification, skin inspection and pain records. To measure the pain intensity, the Numerical Scale was employed and, to assess the risk of developing pressure ulcer in the preoperative phase, the Braden Scale was adopted.

The Braden scale is used to assess the risk of developing pressure ulcer in the global context. This scale consists of six domains: sensory perception, humidity, activity, mobility, nutrition, friction and shear. In this study, the version Paranhos adapted to Brazil in 1999 was employed. The participants were classified according to the Braden scale score for the risk of pressure ulcer, as follows: very high risk (patient scoring nine or less), high risk (patient scoring equal to or between 10 and 12), moderate risk (patient scoring 13 or 14) or at risk (adult patient scoring 15 or 16 and elderly patient scoring 17 or 18)^9^.

Before the data collection, the nurse invited for the inter-rater reliability phase of the scale was trained. Then, a pretest was undertaken with 10 patients (not included in the sample) to adapt the dynamics of the data collection, which happened in the first semester of 2013 and took five months.

The data were collected as follows: preoperative period - after confirming the surgery schedule, the patient was selected based on the inclusion criteria. The researcher visited the patient before the surgery to complete the data in tool 1, skin inspection (first the patient's position was changed and, 30 minutes later, the skin was inspected), registration of Braden scale score and verification of presence of pain (type, place and intensity) using the Numerical Scale.

In the intraoperative period, the researcher and an invited nurse monitored the patient since the entry into the surgery room until the transfer to the post-anesthetic recovery room to register the ELPO score (version 2). In the Postoperative period (PO), the patient's skin was inspected in the Immediate Postoperative period (IPO) and daily until the limit of four days of PO, or until a pressure ulcer appeared (outcome), in case that happened earlier. In addition, the researcher also assessed the pain outcome deriving from the surgical positioning through the application of the Numerical Scale on the first and second PO day.

To calculate the Content Validity Index (CVI), th number of answers to the subitems scored as 4 (I agree) and 5 (I completely agree) was added up for all scale items and divided by the total number of answers the experts had provided^10^.

The statistical tests were developed in the software Statistical Package for the Social Sciences (SPSS), version 21.0, for Mac OS X. Student's t-test was used for the concurrent criterion validity, comparison between the mean ELPO score and the Braden scale categories. For the predictive criterion validity, logistic regression was applied to verify the association of the ELPO with the chance of developing pain outcomes due to surgical positioning and Pressure Ulcer (PU). The ICC was calculated to determine the inter-rater reliability of the tool.

To conduct the study, the research project received approval from the Research Ethics Committee (Protocol 1472/2011), in compliance with Resolution 196/96, and all participants (experts and patients) signed the Informed Consent Form.

## Results

**Figure 1:**
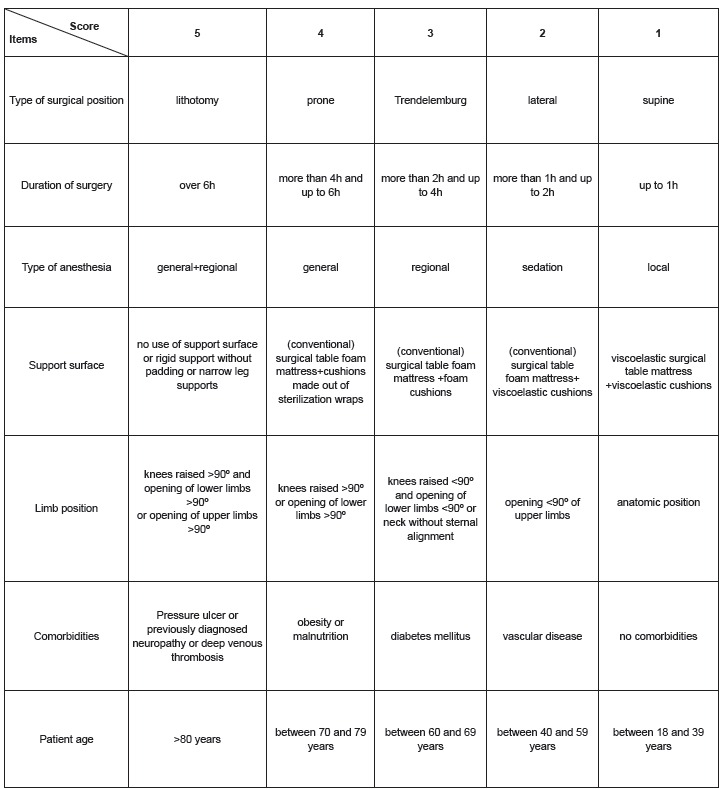
Risk assessment scale for the development of injuries due to surgical positioning (ELPO, version 2)

The CVI calculated for all experts' answers corresponded to 0.88, with a median of 0.96. This result indicated that 88% of the experts considered the ELPO (version 1) a relevant tool to assess the patient risk for the development of injuries due to the positioning. Despite the few changes in version 1 of the ELPO, after the face and content validation phase of the scale, the authors named the scale ELPO (version 2) (Figure 1).

The ELPO (version 2) contains seven items with five subitems each. The score ranges from one to five points and the total score from seven to 35 points. The higher the patient's score, the greater the risk of developing injuries due to surgical positioning.

Among the 115 participants in the field research, 69 (60%) were female, 46 (40%) male, with a mean age of 49.6 years. Most of them (n=73; 63.5%) were employees from the city of Pouso Alegre, Minas Gerais (n=60; 52.2%).

In the preoperative period, 87 patients (75.7%) did not present any type of pain, 99 (86.1%) had no physical limitation and 107 (93%) had no skin injury. The mean Body Mass Index (BMI) in the sample was 25.66kg/m^2^. The mean Braden score was 21.29, indicating no risk for the development of PU.

In the intraoperative period, the most frequent surgical specialty was orthopedics with 23 procedures (20%), followed by 21 (18.2%) neurosurgeries, 19 (16.5%) cardiovascular surgeries, 17 (14.8%) gynecological procedures, 13 (11.3%) general surgeries, 10 (8.7%) plastic surgeries and 12 (10.4%) surgeries in other specialties.

In Table 1, the patient's distribution with regard to the research variables is indicated: duration of surgery, type of anesthesia, type of surgical position, type of support surface and positioning of the patient's limbs in the intraoperative period.

**Table 1:** Distribution of patients from a general hospital according to research variables in the intraoperative period. Pouso Alegre, MG, Brazil, 2013

Variables		n (%)
Duration of surgery (hours)		
	Up to 1	10 (8.7)
	More than 1 up to 2	42 (36.5)
	More than 2 up to 4	36 (31.3)
	More than 4 up to 6	17 (14.8)
	More than 6	10 (8.7)
Type of anesthesia		
	Local	03 (2.6)
	Regional	37 (32.2)
	General	40 (34.8)
	General+regional	35 (30.4)
Type of surgical position		
	Supine	83 (72.2)
	Lateral	04 (3.5)
	Trendelemburg	09 (7.8)
	Prone	10 (8.7)
	Lithotomy	09 (7.8)
Type of support surface		
	Foam mattress + viscoelastic cushions	03 (2.6)
	Foam mattress + foam cushions	24 (20.9)
	Foam mattress + sterilization wrap cushions	69 (60.0)
	No uso of support surfaces or rigid support without padding or narrow leg supports	19 (16.5)
Surgical positioning of the limbs		
	Anatomic position	15 (13)
	Opening <90° of upper limbs	42 (36.5)
	Knee raising <90° and lower limb opening <90° or neck without sternal alignment	30 (26.1)
	Knee raising >90° or lower limb opening >90°	02 (1.7)
	Knee raising >90° and lower limb opening >90° or upper limb opening >90°	26 (22.6)

In the research sample, 42 participants (36.5%) were submitted to surgeries taking more than one and up to two hours. General anesthesia was the most frequent type of anesthesia (n=40; 34.8%). Most patients (n=83; 72.2%) remained in the supine position during the intraoperative period, 69 participants (60%) used the foam mattress and sterilization wrap cushions and 42 patients (36.5%) stayed with their upper limbs open at an angle < 90^0^.

In the postoperative period, out of 115 patients, 46 (40%) presented pain due to the surgical positioning and 25 (21.7%) developed PU. As appointed, to assess the pain intensity, the Numerical Scale was used (self-reported scale ranging between 0 and 10). Score 5 was the most frequent with 13 reports, followed by score 4 indicated by 11 patients (both scores relate to moderate pain).

Among the 25 participants with PU, three already had injuries (stage I), which the researcher had identified in the preoperative period. After the surgical anesthesia, in three cases, the condition evolved to stage II. In two participants, the injuries were identified in the immediate postoperative period and classified as stage I. On the first postoperative day, PU were found in 11 patients (eight injuries classified as stage I and three as stage II). In nine patients, the injuries were identified on the second postoperative day, all of which were classified as stage I.

The mean score on the ELPO (version 2) in the research sample (n=115) was 19.53 (Standard deviation sd=3.85), median 19, minimal score 12 and maximum 30.

To verify the concurrent criterion validity, the mean ELPO scores (version 2) were compared with the Braden score, considering only two groups: the moderate risk/risk group (score between 13 and 18 points) and the group without risk of developing PU (score superior to 19 points), as no patient scoring less than 12 points on the Braden scale was identified in this study (the lowest score was 13 points). Hence, none of the patients was classified as very high and high risk of developing PU. Using Student's t-test, the mean ELPO score for the group at moderate risk or at risk of developing PU (n=14) corresponded to 23.57 (sd=3.47) and, for the group without risk (n=101), to 18.98 (sd=3.56). The mean difference of 4.59 points between the two groups was statistically significant (p<0.001).

In the predictive criterion validity, the comparison of the mean ELPO (version 2) scores between the groups in pain due to the surgical positioning and without pain showed a difference by more than three points (group with pain, higher average), which was statistically significant (p<0.001). In addition, in the logistic regression analysis with a 95% confidence interval, adopting the presence or not of pain as the outcome and the ELPO scores as the predictor, the odds ratio corresponded to 1.28 (OR=1.28), that is, for each additional point on the ELPO, the patient's chance of pain due to the surgical positioning increases by 28%.

The mean ELPO (version 2) score in patients who did not develop PU amounted to 18.55 and, for patients who developed this type of injury, to 23.08, that is, a difference by almost five points between the groups, with statistical significance (p<0.001). The logistic regression analysis indicated an odds ratio of 1.44 (OR=1.44), which means that, for each additional point on the ELPO, the chance of developing PU increases by 44%.

In the comparison of the ELPO (version 2) scores, assessed by the two independent raters (researcher and invited nurse), the minimum, maximum and means were identical between the observers and the variance amounted to 14.81 and 14.58, respectively. Adopting a 95% confidence interval, the ICC corresponded to 0.994 with p<0.001, that is, statistically significant and considered excellent^11^.

## Discussion

In the preoperative phase, the use of assessment scales that include internal and external risk factors for the emergence of injuries can help the nurse to identify patients at higher risk. Through the use of this tool, this professional can plan the implementation of effective solutions in the intraoperative period (e.g. the use of effective pressure relief devices) to prevent the patient from suffering injuries due to surgical positioning^12^.

Based on the international and Brazilian literature, there is a lack of studies on risk assessment scales for the development of injuries due to surgical positioning. Hence, the elaboration of the ELPO was based on recent evidence and its development covered aspects related to different injuries the perioperative nurse can assess. In addition, the selection of the items included in the scale follows expert recommendations on the theme^4^ ^,^ ^6^ ^,^ ^13^ ^-^ ^14^.

The development of this study permitted the construction of a scale the nurses can use to support decision making on care delivery for surgical patients, mainly to prevent possible complications related to the surgical positioning, and also permitted the assessment of the metric properties of the ELPO (concurrent and predictive criterion validity and inter-rater reliability).

The comparison between the CVI calculated for the ELPO with another recent study^15^ revealed a consensus among the health professionals who participated in the expert committee about what the ELPO is intended to measure. In addition, the tool seems to address the content area that is being measured.

The concurrent criterion validity was verified between the Braden and ELPO scores. Patients at moderate risk or at risk of developing PU both presented higher ELPO scores, indicating an increased risk for the development of injuries due to the surgical positioning, particularly PU.

To assess the predictive criterion validity in this study, the types of injuries investigated in relation to the patient's surgical positioning were the presence of pain^6^ ^)^ and the development of PU^9^ ^,^ ^14^ ^,^ ^16^. The results indicated that higher ELPO scores predict the presence of pain and the occurrence of PU, that is, patients with higher ELPO scores have a greater chance of presenting pain and developing postoperative PU due to the surgical positioning.

In that sense, perioperative nurses' application of the ELPO to adult patients can support nursing care planning, guiding intraoperative actions to prevent pain due to the surgical positioning and PU.

The inter-rater reliability analysis demonstrated almost identical results for the two observers in the application of the ELPO. The reliability or trustworthiness of a quantitative measure is one of the main criteria to assess its exactness^11^.

### Recommendations for the use of ELPO in clinical practice

The ELPO is a simple scale that is easy to apply. To use it, the nurses should be familiar with its items and subitems in order to speed up the registration of the scores during its intraoperative application.

The ELPO should be applied when the patient is positioned on the surgery table; in scoring each item, the highest score corresponding to the item should be considered, for example, if the patient was submitted to local anesthesia and sedation, his classification under sedation is recommended, receiving score 2 on the scale.

The item duration of the surgery should be estimated, so that care with the positioning is executed, and should be reassessed at the end of the surgery and correctly classified. If the patient needs to be repositioned during the procedure, the ELPO should be applied again and the full length of the surgery during which the patient remained in each surgical position should be considered.

In clinical practice, the implement the ELPO as a tool to guide nursing decision making on the best care for surgical patients related to positioning, its gross score should be used. Nevertheless, to further the dynamics in clinical practice and facilitate the development of institutional protocols, a cut-off point was suggested for patient risk classification. Therefore, the ROC-curve (Receiver-Operating Characteristic*)* was applied and, according to the result, score 20 is the cut-off point to distinguish patients classified using the ELPO, that is, patients scoring up to 19 points can be classified as lower risk for the development of injuries due to surgical positioning, while patients scoring 20 or higher can be classified at higher risk.

Based on the above classification, it can be established which patients the health professionals should be more cautious with during the surgical positioning, in order to prevent complications associated with the procedure. In addition, spending on the patient can be justified, like in the case of a support surface or an additional cost.

To enhance patient care during surgical positioning, educative programs for nursing, anesthesia and surgical teams are recommended, addressing the best practices for this procedure, including a preoperative visit to get to know each patient's particularities and provide for the support devices and surfaces needed for the surgical positioning the patient will remain in during the intraoperative period.

Concerning the study limitations, the application of the ELPO was restricted to a single hospital. Therefore, further research is needed to assess its use in clinical practice, which will offer support to verify whether the use of this tool can promote positive results in the prevention of injuries due to surgical positioning. Furthermore, the constant search for evidence and the periodical review of the ELPO items are important aspects thanks to knowledge advances and technological development, mainly regarding pressure relief devices used in the intraoperative period.

## Conclusion

The ELPO is a valid and reliable tool to assess the risk for the development of injuries due to surgical positioning in adult patients. The assessment of its use in clinical practice depends on further research in different hospital contexts.

In clinical practice, the application of the ELPO can support nursing decision making on patient care, during the surgical positioning, promoting improvements in nursing care and stimulating the development of nursing care protocols for the surgical positioning of patients.

## References

[1] Landi A, Corradetti E, Mancarella C, Delfini R (2013). Prevention of complications related to patient prone positioning
during spinal neurosurgical care a nursing point of view. J Spine Neurosurg.

[2] Beckett AE (2010). Are we doing enough to prevent patient injury caused by positioning
for surgery. J Perioper Pract.

[3] Spruce L, Van-Wicklin SA (2014). Back to basics positioning the patient. AORN J.

[4] Association of periOperative Registered Nurses (2015). Guideline for positioning the patient. In Guidelines for perioperative
practice, 2015 Edition.

[5] Barbosa MH, Oliva AMB, Sousa AL (2011). Ocorrência de lesões perioperatórias por posicionamento
cirúrgico. Rev Cubana Enferm.

[6] Menezes S, Rodrigues R, Tranquada R, Muller S, Gama K, Manso T (2013). Lesões decorrentes do posicionamento para cirurgia incidência e
fatores de risco. Acta Med Port.

[7] Navarro-Vicente F, García-Granero A, Frassom M, Blanco F, Flor-Lorente B, García-Botelho S (2012). Prospective evaluation of intraoperative peripheral nerve injury in
colorectal surgery. Colorectal Dis.

[8] Rogenski NMB, Kurcgant P (2012). Measuring interrater reliability in application of the Braden
Scale. Acta Paul Enferm.

[9] Ayello EA. (2012). Predicting pressure ulcer risk.General assessment series: try this: issue
5.

[10] Alexandre NMC, Colluci MZ (2011). O. Validade de conteúdo nos processos de construção e adaptação de
instrumentos de medidas. Ciênc Saúde Coletiva.

[11] Alexandre NMC, Gallasch CH, Lima MHM, Rodrigues RCM. (2013). A confiabilidade no desenvolvimento e avaliação de instrumentos de
medida na área da saúde. Rev Eletr Enferm.

[12] Bouyer-Ferullo S (2013). Preventing perioperative peripheral nerve injuries. AORN J.

[13] Munro CA (2010). The development of a pressure ulcer risk-assessment scale for
perioperative patients. AORN J.

[14] Primiano M, Friend M, Mcclure C, Nardi S, Fix L, Schafer M (2011). Pressure ulcer prevalence and risk factors during prolonged surgical
procedures. AORN J.

[15] Joventino ES, Oria´ MOB, Sawada NO, Ximenes LB (2013). Apparent and content validation of maternal self-efficiency scale for
prevention of childhood diarrhea Rev. Latino-Am. Enfermagem.

[16] Nilsson UG (2013). Intraoperative positioning of patient under general anesthesia and the
risk of postoperative pain and pressure ulcer. J Perianesth Nurs.

